# The burden of headache disorders in Pakistan: methodology of a population-based nationwide study, and questionnaire validation

**DOI:** 10.1186/1129-2377-14-73

**Published:** 2013-08-22

**Authors:** Arif D Herekar, Akbar A Herekar, Ali Ahmad, Umer L Uqaili, Bilal Ahmed, Jahanzeb Effendi, Syed Z Alvi, Timothy J Steiner

**Affiliations:** 1 Department of Neurology, Baqai Medical University, Karachi, Pakistan; 2 Department of Neurosurgery, Dow University of Health Sciences, Karachi, Pakistan; 3 Department of Surgery, Dow University of Health Sciences, Karachi, Pakistan; 4 Department of Medicine, Dow University of Health Sciences, Karachi, Pakistan; 5 Department of Neuroscience, Norwegian University of Science and Technology, Trondheim, Norway

**Keywords:** Migraine, Tension-type headache, Medication-overuse headache, Burden of disease, Epidemiology, Population-based survey, Pakistan, Global Campaign against headache

## Abstract

**Background:**

Large geographical gaps in our knowledge of the prevalence and burden of headache disorders include Pakistan, a country with major problems of poverty, illiteracy and security. We report implementation in this country of standard methods developed by *Lifting The Burden* (LTB) for population-based burden-of-headache studies.

**Methods:**

We surveyed six locations from the four provinces: Lahore and Multan (Punjab), Karachi and Sukkur (Sindh), Abbottabad (Khyber Pakhtunkhwa) and Gwadar (Baluchistan). We randomly selected rural and urban households in each, which were visited by trained non-medical interviewers from the same locations. One randomly selected adult member (18–65 years) of each household was interviewed using LTB’s structured questionnaire translated into Urdu, the national language. Validation was performed among patients and accompanying attendants in three (urban and rural) medical facilities. After responding to the questionnaire, these participants were re-interviewed and diagnosed by a neurologist (gold standard).

**Results:**

The survey was completed by 4,223 respondents (1,957 [46.3%] male, 2,266 [53.7%] female, 1,443 [34.2%] urban, 2,780 [65.8%] rural, mean age 34.4 ± 11.0 years). The participation rate was 89.5%. There were 180 participants (46.1% male, 53.9% female, 41.7% urban, 58.3% rural, mean age 39.4 ± 14.2 years) in the validation sample, of whom 147 (81.7%) reported headache in the last year. The questionnaire was 100% sensitive in screening for headache and for headache on ≥15 days/month, and showed good agreement with the gold-standard diagnoses (kappa = 0.77). It was relatively insensitive for TTH. The questionnaire’s default diagnosis of probable MOH when medication overuse accompanied headache on ≥15 days/month was not supported by evidence of causation in most cases seen by the neurologist. In public-health terms, precise diagnosis in these cases matters less than reliably detecting the coexistence of these disorders.

**Conclusion:**

In conclusion, the methods developed by LTB were applied successfully in Pakistan, despite problems unique to this country.

## Background

Headache disorders are amongst the most common complaints. Although disabling, they remain under-recognized and under-treated throughout the world [1]. The Global Burden of Disease study 2000 (GBD2000) included migraine among the diseases surveyed, and found it to be the 19th highest cause of disability in the world, contributing 1.4% of all years lost to disability (YLDs) [2]. At the time of this survey, epidemiological data were lacking on headache in very large parts of the world, including China, India, most of Africa and all of the Eastern Mediterranean Region. The Global Campaign against Headache [3,4], conducted by the UK-registered non-governmental organization *Lifting The Burden* (LTB) in official relations with the World Health Organization (WHO) [5], made its priority to fill these gaps [6-8]. An important consequence was that the Global Burden of Disease study 2010 (GBD2010) was much better informed, and able to include tension-type headache (TTH) as well as migraine. In this survey, migraine was ranked 7th highest among specific causes of disability [9,10], responsible for 2.7% of all YLDs in the world.

In the Eastern Mediterranean, studies were initiated in Pakistan and Saudi Arabia, and are planned in Morocco and Egypt. Pakistan, although a country with over 180 million people, had remained untouched as far as knowledge of the prevalence and burden of headache disorders was concerned: to the best of our knowledge, no local data for the general population were available. Few population-based studies existed for developing countries, where limited funding and large rural populations make difficult the systematic collection of information. Coupled with the low profile of headache disorders compared with other diseases, these factors are a significant deterrent to such studies. Further, given the high poverty level and low level of literacy in Pakistan, people disabled by headache might, as a rule, not seek medical help. The consequence would be that the public ill health caused by headache disorders remained concealed.

The purpose of this study was to fill this knowledge gap in Pakistan, while contributing to estimates of the burden of headache disorders in the Eastern Mediterranean Region. Gathering this knowledge is not an end in itself: to estimate the prevalence and burden of headache in Pakistan is a necessary first step in planning and implementing appropriate measures to reduce the burden. These measures require political will and support; the knowledge from this study will make the case to health authorities and government that a major public-health priority has been ignored, with very large humanitarian and socioeconomic costs [1].

Methodologically, the study built on a protocol developed for and experience gained in previous similar studies conducted at the instigation of LTB [6-8]. The adaptation of these methods for Pakistan and validation of the diagnostic questionnaire are described here.

## Methods

### Ethics

The Ethics Review Board at the Dow University of Health Sciences, Karachi, approved the study. All respondents gave written consent prior to interview, and had the option of discontinuing the interview at any time during it.

Data protection laws were complied with. No information relating to identifiable individuals circulated beyond the researchers immediately involved in the study.

### Population of interest

We aimed to study the adult general population (aged 18–65 years) resident in Pakistan.

### Study design

The design that would best achieve our objectives was a cross-sectional community-based survey, employing cluster sampling.

The enquiry procedure involved unannounced door-to-door visits at households (“cold-calling”) within each cluster, and application by trained interviewers of a structured questionnaire.

### Study questionnaire

We used the questionnaire developed by LTB for a similar study in India [7], with minor changes for a Pakistani population. It was in six sections. The first covered basic demographic and socio-economic details including age, gender, income and occupation. The second enquired into occurrence of any headache in the last year (screening): “Have you had a headache in the last year?” Those who responded “yes” were then asked the remainder of the questions. The third section was diagnostic, distinguishing between episodic headache and headache occurring on ≥15 days/month and using modified ICHD-II criteria [11] for migraine, TTH and medication-overuse headache (MOH). This section first asked whether all headaches were of the same type; if not, the respondent should focus on the type that was subjectively the most bothersome. The fourth enquired into point prevalence (headache yesterday). The fifth focused on burden attributable to headache, including disability measured as lost productive time (the HALT index [12]) and health-care utilization. The last, applied to all respondents, with or without headache, was on quality of life (WHOQoL-8 [13]).

### Pre-pilot study

To establish that the questions would be comprehensible, acceptable and inoffensive to respondents, and the questionnaire usable, we conducted a pre-pilot study in Karachi. This city, the largest and most ethnically diverse in Pakistan, is inhabited by a large percentage of migrants from all over the country. The venues were the neurology outpatients’ department of the Civil Hospital, Karachi, and the Neurodiagnostics Centre, Karachi. We administered the questionnaire to 100 respondents, a mix of patients and their attendants, translating the questions at the point of application.

### Translation

After this study, we employed LTB’s protocol for lay documents [14] to translate the questionnaire into the national language, Urdu.

### Sampling and enquiry

We conducted the study in all four provinces of Pakistan: Sindh, Punjab, Balochistan and Khyber Pakhtunkhwa (previously North-West Frontier Province).

### Locations

We chose six study locations from the four provinces: Punjab (Lahore and Multan) and Sindh (Karachi and Sukkur) were allocated two locations each because of their larger populations. The others were Abbottabad for Khyber Pakhtunkhwa and Gwadar for Baluchistan. We defined urban settings as within city limits (which included suburban areas) and rural settings as outside these limits. We selected rural areas randomly, but keeping within a 50-km radius outside city limits: this was necessary for reasons of security, time available for the study and cost and feasibility of transport.

### Interviewers

We appointed 12 non-medical interviewers recommended by the investigators of a recently-conducted study on infectious diseases in Pakistan, selecting them from the different study regions: four each from Karachi and Lahore and two each from Multan and Abbottabad (Sukkur and Gwadar were covered by the Karachi interviewers). All were fluent in English and Urdu, and had sound knowledge and experience of conducting both medical and non-medical field studies. Belonging to their regions, they were also fluent in the local languages and familiar with local customs and sentiments.

At a 2-day training session held in Karachi, conducted in Urdu, the interviewers were guided through the questionnaire step by step, with special attention paid to important sections (*eg*, the screening and diagnostic questions). Any queries were resolved. On the second day, they were observed and evaluated by the co-investigators using mock interviews.

### Sampling technique

For urban sampling, we obtained a detailed map of each city and its environs from the Federal Bureau of Statistics, and noted the city limits from up-to-date Google maps of these cities. We highlighted the inhabited areas on these maps, excluding unpopulated areas. We divided cities into zones of 5 km^2^ (Karachi, Lahore) or 2 km^2^ (Multan, Sukkur, Abbotabad, Gwadar), and each zone into clusters of 1 km^2^. We numbered the zones and clusters, and used a random-number generator to select one zone and, from it, one cluster. Enlarged copies of the maps showing each cluster were given to the interviewers with instructions about the cluster boundaries.

For rural sampling, we made combined use of larger provincial maps and Google maps, supplementing these with information from the local interviewers as the former were somewhat outdated. After carefully demarcating the administrative city limits (within which were urban and suburban areas), we numbered the villages within a 50-km radius outside the city limits and used the random-number generator to select an adequate number of these.

In each cluster (urban) or village (rural), the interviewer selected households by writing house numbers on chits and randomly picking from these. Commercial buildings and those offering paying-guest accommodation (hotels, hostels, *etc.*) were excluded. Cold-calling at households was timed so that probability of finding household occupants at home was maximized; since most females in rural areas work in the morning hours, rural households were approached in the afternoons. When there was no answer, the interviewers returned after a few hours. When the second visit was also unsuccessful, a new household was selected from the remaining chits. On entry, the interviewer explained the study’s purpose and nature. All adults aged 18–65 years were listed, and one randomly selected by the interviewer, again by drawing one from a bowl of numbered chits. If that person was available, the interview was commenced immediately; otherwise, an appointment was made for the interview to be conducted at a later time on the same day.

If the chosen person was unable to communicate because of mental incapacity or other illness, another was selected. Otherwise there were no replacements at household level.

### Sample size

We planned a total sample size of 4,149. We assumed, without guiding data, a headache prevalence of 0.5 (50%) and applied a confidence level of 99% and confidence interval of 2% in calculating the necessary sample size.

We divided this sample between the study locations in accordance with population estimates for 2006 stated in the 1998 census [15], this being the most recent (the 2011 census still undergoing analysis). The allocations were: Punjab 57.5% (Lahore 38.5%; Multan 19%); Sindh 24% (Karachi 22%; Sukkur 2%); Baluchistan 5%; Khyber Pakhtunkhwa 13.5%. Within each location we divided the sub-samples between urban and rural areas using the city:urban ratio reported for each in the 1998 census [15].

### Quality assurance

The quality of the data was considered of paramount importance. We took multiple steps to ensure it.

Responsibility for the study in the six locations was divided among the six co-investigators. A chain of command was constructed in which the co-investigators were responsible for communication with interviewers, collection and transport of data, disbursement of interviewers’ salaries and study location review visits at the end of data collection.

After their training session, interviewers proceeded to their respective study locations, each with a personalized package including a bag, weighing scale, measuring tape, white coat, location map and picture identification card. From each of the six interviewer pairs, one was appointed as location supervisor and directly reported to the assigned co-investigator. The supervisor was responsible for timely collection of data and their safe transport from the study location to base. Weekly updates were provided to all co-investigators and any problems faced by the interviewers in the administration of the interviews or logistics were dealt with.

Two thirds of the way through data collection, the co-investigators as a team visited each study location for review. Questionnaires representing 10% of the location sample were randomly selected and the respective households were revisited to determine the authenticity of the data. Once this had been established, the study was completed.

A major discrepancy was encountered at Multan. On re-visiting households, the co-investigators found very few of the randomly selected questionnaires to be authentic. Analysis of the data from Multan showed they were unfeasibly different from those from all other locations. The details of detection and rectification of this fraud will be presented elsewhere. The entire study in Multan was repeated with two new interviewers under legally-binding contract. At the completion of data collection, a co-investigator visited the location for review, and found the data to be authentic.

### Diagnosis

#### Diagnostic algorithm

Diagnoses were not made by the interviewers, but subsequently by diagnostic algorithm applied to the most bothersome headache. Experience has shown that questionnaires cannot distinguish reliably between headache disorders characterized by headache on more days than not (*eg*, chronic migraine, chronic TTH), but can identify presumptive MOH from the reported frequency and type of medication taken for the headache. Therefore, cases were removed for individual review of medication use when headache was reported on ≥15 days/month, and diagnosed either as probable MOH or “other headache on ≥15 days/month”. All remaining cases (episodic headache) were sorted by applying ICHD-II criteria in hierarchical sequence: first definite migraine (dMIG), then definite TTH (dTTH), then probable migraine (pMIG) and finally probable TTH (pTTH). Cases falling into none of these categories were “undetermined”. During subsequent analysis, dMIG and pMIG were combined (allMIG), as were dTTH and pTTH (allTTH) for generating prevalences.

#### Validation study

The validation study was carried out in part in the neurology outpatient department of a tertiary care hospital in Karachi, in part in Delhi colony, an urban residential area in Karachi where a free medical camp had been set up, and in part in a rural medical camp near Hyderabad, in the province of Sindh. In this way we interviewed a diverse group of respondents representing both rural and urban populations. They were a mix of patients reporting headache as their primary complaint and their accompanying attendants, who were not patients. This method of convenience sampling did not conform to the random population sampling of the main study, but was the best possible compromise, enriching the sample with people with troublesome headache while including a more representative subset of non-patients. Respondents were recruited consecutively, unless meeting one or more of the exclusion criteria: age below 18 or over 65 years; headache apparently due to another disorder (other than medication overuse) (the questionnaire did not have the ability to diagnose secondary headache); mental incapacity; non-consent.

First, the study questionnaire was administered by one of the co-investigators, who were medical students at the time, unable to support the questions with expert knowledge. Then each respondent was seen by a neurologist and headache expert (AH), sitting in another room within the facility, ignorant of the questionnaire responses. He applied a combination of ICHD-II criteria [11] and his professional expertise to make a diagnosis, which would be taken as the “gold standard” against which questionnaire diagnosis was compared. Those who responded “no” to the screening question were also seen by the neurologist.

### Data management

At the base centre, returned questionnaires were reviewed. Those that were unusable because they were incompletely or inaccurately filled were excluded. Data from the accepted questionnaires were entered into SPSS version 16.0. We applied full double data-entry by two key-punch operators working independently, subsequently comparing databases to remove errors after reference to the original forms. In addition, approximately 10% of the data were randomly cross-checked against the original forms by the co-investigators, finding minimal discrepancies.

### Statistical analysis

We used descriptive statistics (mean ± standard deviation) to characterize samples.

Definite and probable questionnaire diagnoses were combined in the validation study. Sensitivity, specificity and positive (PPV) and negative (NPV) predictive values of the questionnaire for the different diagnoses (migraine, TTH, MOH), along with kappa values, were calculated using PASW Statistics version 18.0.

## Results

### Main study

The survey was completed for 4,223 respondents (1,957 [46.3%] male and 2,266 [53.7%] female) aged 18–65 years (mean 34.4 ± 11.0), of whom 1,443 (34.2%) lived in urban and 2,780 (65.8%) in rural areas. The participation rate was 89.5% overall, with regional variation between >99% and 69%. In rural areas of Lahore, the refusal rate was 31.0% without reason being offered, and 11 interviews were terminated after they had been started. In Karachi, a total of 12 only refusals were encountered out of 950 contacts. Multan, Abbottabad, Sukkur and Gwadar all had refusal rates of <1% but, in Gwadar, there were two incidents in which the interviewers themselves felt uncomfortable after the interviews had started and terminated them.

Table 1 shows the demographic characteristics of the total sample, and comparisons with 2008/09 extrapolated figures from the Federal Bureau of Statistics (FBS) for all Pakistan. These showed that, in our sample, males and the over-50 age groups were slightly under-represented.

**Table 1:** **Demographic characteristics of the total sample and comparisons with national statistics (extrapolated to 2008/9 from 1998 census) from the Federal Bureau of Statistics (FBS)**[15]

**Variable**	**Sample n (%)**	FBS statistics (%)*
**Gender**		
Male	1,957 (46.3)	49.7
Female	2,266 (53.7)	50.3
**Age group (years)**		
18-29	1,507 (35.7)	36.4
30-39	1,197 (28.3)	25.4
40-49	1,101 (26.1)	20.5
50-59	330 (7.8)	13.1
60-65	88 (2.1)	4.6
**Marital status**		
Married	3,015 (71.4)	
Unmarried	1,089 (25.8)	
Separated, divorced or widowed	78 (1.8)	
Unknown	41 (1.0)	
**Habitation**		
Rural	2,780 (65.8)	66.4
Urban	1,443 (34.2)	33.6
**Education**		
None	165 (4.0)	
School	2,977 (71.4)	
College	1,027 (24.6)	

Pakistan, 2013.

*All FBS statistics were recalculated for the population aged 18–65 years.

A few specific hurdles were encountered during administration of the questionnaire. A significant proportion of people residing in rural areas earned rent on their ancestral lands, but were recorded as “unemployed”. A majority of participants were not comfortable with disclosing their annual income for security reasons. Many, when asked the names of medication used for their headaches, were unable to give definite answers because of the prevailing illiteracy in the general population of the country.

### Validation study

A total of 180 participants were interviewed (mean age 39.4 ± 14.2 years; male 46.1%, female, 53.9%; urban 41.7%, rural 58.3%). No potential respondents withheld consent. Of the 180, 147 (81.7%) reported headache in the last year.

There was 100% concordance between questionnaire and neurologist over the 33 (18.3%) cases with no headache. Among the remaining respondents, the questionnaire diagnosed 47 (26.1%) cases of migraine, 36 (20.0%) of TTH, 42 (23.3%) of probable MOH and 22 (12.2%) of other headache on ≥15 days/month. No cases were unclassified. The neurologist diagnosed 38 (21.1%) cases of migraine, 42 (23.3%) of TTH, 7 (3.9%) of probable MOH and 58 (32.2%) of other headache on ≥15 days/month, with 2 (1.1%) cases undetermined (mixed headaches). Table 2 compares these diagnoses, and Table 3 gives sensitivities, specificities, PPVs and NPVs for each headache type. The kappa value for overall agreement between questionnaire and neurologist was 0.59; individual kappa values for migraine, TTH and MOH were 0.56, 0.54 and 0.19 respectively. When MOH and other headache on ≥15 days/month were combined, kappa values for migraine, TTH and all headaches on ≥15 days/month were 0.56, 0.54 and 0.98 and overall kappa was 0.77.

**Table 2:** Cross tabulation of questionnaire and neurologist’s diagnoses

**Questionnaire diagnoses**	**Neurologist’s diagnoses**						
	**No headache**	**Migraine**	**TTH**	**Probable MOH**	**Other headache on ≥15 days/month**	**Undetermined**	**Total**
**No headache**	33	0	0	0	0	0	33
**Migraine**	0	28	17	1	0	1	47
**TTH**	0	10	25	0	0	1	36
**Probable MOH**	0	0	0	6	30	0	36
**Other headache on ≥15 days/month**	0	0	0	0	28	0	28
**Undetermined**	0	0	0	0	0	0	0
**Total**	33	38	42	7	58	2	180

Pakistan, 2013.

**Table 3:** Sensitivities, specificities, positive predictive values (PPVs), negative predictive values (NPVs) and Kappa values for questionnaire diagnoses compared with “gold standard”

	**Sensitivity**	**Specificity**	**PPV**	**NPV**	**Kappa**
No headache	1	1	1	1	
Migraine	0.74	0.87	0.60	0.92	0.56
TTH	0.60	0.92	0.69	0.88	0.54
Headache on ≥15 days/month	0.98	1	1	0.99	0.98
Probable MOH	0.86	0.82	0.17	0.99	0.19

Pakistan, 2013.

## Discussion

This study was the first nationwide survey of headache disorders performed in Pakistan. As in all such studies, meticulous methods and a reliable diagnostic instrument were crucial requirements. Here we present our methodology and validation of the diagnostic questionnaire, both adapted from those used by LTB in similar studies in India [7], Russia [8] and China [6].

These were the limitations, and how we responded to them. Conducting a door-to-door survey throughout Pakistan, a developing country beset by security issues, was a logistic challenge. From the initial stages onwards, very careful planning and circumspection were applied in order to anticipate and pre-empt problems. Some of our difficulties had been faced by other studies conducted in similar regions [7]. However, these and others stood in sharp contrast to those of the western world: widespread illiteracy, lack of infrastructure, multiple languages, adherence to different tribal cultural values, corruption and ethnicity-based violence. All these factors had to be overcome as far as was possible. In addition, regarding headache prevalence and burden, Pakistan was uncharted territory. In these points lies the significance of our study.

Poor infrastructure is always a barrier to conducting geographically widespread surveys in developing countries, and was no less so in Pakistan. Nevertheless, we invested the high cost and effort demanded by a door-to-door survey in order to reach a nationally-representative, sample. We were forced into one compromise: when sampling rural areas, we chose locations within a 50-km radius from the city limits. Although this meant that the populations we sampled had more access to health care than others more distant from the cities, it contained costs somewhat and, more importantly, protected the security of our interviewers. It also facilitated monitoring and quality control (discussed later). The security situation was a major hindrance, unique to this country among those surveyed by LTB. Sindh and Punjab, the two heavily-populated and safer provinces, were adequately represented. However, to prevent any untoward incident, Balochistan and Khyber Pakhtunkhwa – less populated and less secure – were sampled more selectively, from their more accessible parts. In a second compromise, in most cases we limited revisits to unresponsive households to one, later on the same day. Travelling to distant locations was costly in time and money, and we judged that revisiting them in order to interview only a few households (interviewers reported between one and five, depending on the area) would have been a significant waste of resources. Analysis showed that our sample was fairly representative when compared with FBS national population statistics, themselves extrapolated forwards 10 years from the 1998 census.

We believe it unlikely that these two methodological compromises were significantly detrimental to the quality of the study.

Keeping in mind the diversity of the population in different parts of the country, we trained interviewers from their own regions. Since they spoke the local languages, and understood the local cultures and sentiments, this undoubtedly promoted willingness to participate, with refusal rates in most areas of <1% of those approached. Exceptions were the rural areas of Lahore (31.0%) and urban areas of Karachi (2.7%). We began the survey in these two locations a few days earlier than in the others, and learned from problems encountered here. In rural Punjab, many of those who were illiterate refused to be interviewed without the permission of the religious head of the village or district first. Interviewers were subsequently instructed to seek permission from the religious or political heads before approaching such areas. In terror-stricken urban Karachi, reasons for refusal varied from ethnic (a household of a certain ethnicity would not engage with an interviewer from a different ethnic group) to security concerns. We also issued white coats to interviewers, considered a symbol inviting respect.

We aimed for stringent quality control. Interviewers were required to provide weekly updates, problems were dealt with as they were reported, and reviews at each location entailed revisiting 10% of households, randomly selected, to determine authenticity of the data. Yet the experience in Multan demonstrated that epidemiological studies are as vulnerable to fraudulent invention of data as are clinical trials. This experience (which we rectified) will be described as an object lesson in more detail elsewhere.

We validated our diagnostic questionnaire in a sample of 180 respondents from an urban residential area of Karachi and a rural area near Hyderabad. We recognized that validation would ideally have been performed in a sample from the population in which the main study was to be conducted, but not all ideals are realisable in current-day Pakistan. There were benefits to this approach: the inclusion of non-patient accompanying persons broadened the sample, although arguably these attendants were conditioned – perhaps sensitized to the impact of headache – if they were living, as partners or children, with headache patients. The neurologist was able to diagnose the patients face-to-face rather than over the phone, as was done in Russia [8] because of the constraints imposed by enormous geographical spread. The time interval between administration of the questionnaire and diagnosis by the neurologist was only a few hours, eliminating both the recall bias and possibility of change in the condition that are present when the neurologist sees respondents up to a month later. An obvious consequence of sampling from clinical settings was that cases of headache on ≥15 days/month were very many (35.6%), even though 33 of the accompanying persons did not have headache at all. This was not entirely a bad thing: it allowed us, unusually, to assess diagnostic capability for MOH (but see below). The questionnaire was administered in the validation exercise by the medical student co-investigators, and not by the lay interviewers of the main study, but their clinical knowledge of headache was limited and they followed the same scripted protocol for administration of the fully structured questionnaire. ICHD-ll separately codes definite migraine (dMIG) and probable migraine (pMIG), and definite TTH (dTTH) and probable TTH (pTTH). We followed these distinctions at first instance when we applied the algorithm to make diagnoses based on questionnaire responses, but combined definite and probable cases of migraine (allMIG) and of TTH (allTTH) for purposes of comparison with neurologist diagnoses. We agreed with Yu *et al.*, who deemed this “practical and pragmatic” [6], and similarly with Ayzenberg *et al.*[8]. “Probable” diagnoses serve a purpose in clinical management pending confirmation during follow-up, but none in epidemiological surveys (in which all diagnoses are and remain probable).

Overall agreement between the questionnaire and neurologist’s diagnoses was good (kappa = 0.77) when all cases of headache on ≥15 days/month (MOH or otherwise) were combined. We achieved good specificity (87%) and rather lower sensitivity (74%) for migraine, the latter comparable to that in Russia (77%) [8] and higher than that in India (63%) [7], similar studies using the same instrument. With kappa = 0.56 (moderate agreement [16]), the Urdu version of LTB’s questionnaire can be administered with reasonable confidence to ascertain the prevalence of migraine in a population-based survey. Despite lower sensitivity (60%), there was moderate agreement (kappa = 0.54) for TTH also. Other users of the same questionnaire have reported relatively low sensitivities for TTH: Ayzenburg *et al.* recorded 64% [8]. Other questionnaires for the diagnosis of TTH are rare, but Rasmussen *et al.* reported a sensitivity of only 43% (albeit with 96% specificity) [17]. All agree that the reason lies more in the lack of specific characteristics of TTH [11] than in the questions aiming to diagnose it.

There were greater problems with MOH. The questionnaire and neurologist were in almost complete agreement in diagnosing headache on ≥15 days/month, but 30 cases diagnosed by questionnaire as probable MOH were not so recognised by the neurologist. The questionnaire could only record associations in individual participants between headache on ≥15 days/month and current medication overuse (hence probable MOH), applying a threshold for the latter of ≥4 days/week in recognition that drugs for headache consumed by the majority were simple analgesics (acetaminophen, aspirin or other NSAIDs) rather than opioids, ergots or triptans. The neurologist could and did enquire into the ICHD-II criterion: *Headache has developed or markedly worsened during medication overuse*[11]. In fact this criterion has been dropped from the recently published ICHD-3 beta [18] because it was considered unreliable, putting the “gold standard” of our study in doubt. Arguably, from the points of view both of health-care needs assessment and of public education, precise diagnosis does not greatly matter: medication overuse in association with headache on ≥15 days/month is a bad combination whether causation is evident or not. Management in either case, having recognised medication overuse, must focus on strongly discouraging it.

It is worth noting that this issue has not arisen in previous similar LTB studies because few cases of headache on ≥15 days/month have been included in the validation sub-samples selected randomly from the population of interest. Here, of course, more than one third of the validation sample had headache on ≥15 days/month.

## Conclusions

The methods developed by LTB for population-based burden of headache studies, and used in multiple countries and cultures, have been applied successfully in Pakistan, despite problems unique to this country. The Urdu version of the diagnostic questionnaire was 100% sensitive in screening for headache and, importantly, for headache on ≥15 days/month. It showed good agreement with the neurologist’s gold-standard diagnoses, with an overall kappa value of 0.77, being relatively insensitive for TTH. This reflects a general problem: the lack of distinctive features of TTH. The questionnaire’s default diagnosis of probable MOH when medication overuse accompanied headache on ≥15 days/month was not supported by evidence of causation in most cases seen also by the neurologist. In public-health terms, precise diagnosis in these cases matters less than detecting the coexistence of these disorders.

## Competing interests

TJS is a director and trustee of *Lifting The Burden*. He and the other authors report no other competing interests.

## Authors’ contributions

ADH and TJS conceived the study. ADH oversaw the conduct of the study as principal investigator and was the neurologist providing the gold standard for diagnosis. AAH, AA, UL and BA helped design the study, participated in its conduct and coordination and performed the statistical analysis. JE and ZA participated in study design, conduct and data collection. TJS led the design of the study and provided external oversight. All authors were involved in drafting the manuscript and read and approved the final version.
